# Supplementary material for: Structural basis for terminal loop recognition and stimulation of pri-miRNA-18a processing by hnRNP A1
Source: Nat Commun. 2018 Jun 26;9:2479. doi: 10.1038/s41467-018-04871-9 (PMC6018666; doi:10.1038/s41467-018-04871-9)
Supplement: Supplementary file 1 — Supplementary Information [file 41467_2018_4871_MOESM1_ESM.pdf]

Supplementary information

Structural basis for terminal loop recognition and stimulation of pri-miRNA-18a processing by hnRNP A1

Kooshapur et al.

Supplementary Table 1

Effects of hnRNP A1 mutations on subcellular localization and pri-mir-18a processing

**Construct**	**Sequence**	**Localization**	**Processing of pri-mir-18a**
hnRNP A1	RRM1-RRM2-Gly-rich	Nuclear	**++**
UP1-M9	RRM1-RRM2-M9	Nuclear	**++**
UP1-M9_FD2	RRM1-RRM2-M9 **F148D/F150D**	Nuclear	**-**
UP1-M9_FD12	RRM1-RRM2-M9 **F57D/F59D/ F148D/F150D**	Nuclear	**-**
UP1-M9_FD12a	RRM1-RRM2-M9 **F17D/F57D/F59D/ F108D/F148D/F150D**	Mostly nuclear (some in the cytoplasm)	**-**
UP1-M9_FA12b	RRM1-RRM2-M9 **F17A/F57A/F59A/ F108A/F148A/F150A**	Nuclear (almost exclusively)	**-**
RRM1-M9	RRM1-M9	Mostly nuclear	**-**
RRM2-M9	RRM2-M9	Mostly nuclear	**-**

Mutations where phenylalanines are replaced by aspartate residues in RNP-1 motifs of RRM1 and/or RRM2 are denoted FD12 and FD2, respectively (mutations indicated with red font). In FD12a and FA12b, six Phe residues (four in RNP-1 and two in RNP-2 motifs of RRM1 and RRM2) are replaced by Asp and Ala, respectively.

Supplementary Table 2

Small-angle X-ray/neutron scattering (SAXS/SANS) statistics

	SAXS				SANS	
Data collection	UP1	pri-mir-18a	UP1/pri-mir-18a complex	UP1/12-mer complex	UP1/pri-mir-18a complex (0% D_2O)	UP1/pri-mir-18a complex (42% D_2O)
Instrument	X33, DESY, Hamburg	X33, DESY, Hamburg	X33, DESY, Hamburg	Rigaku BioSAXS-1000	D22, ESRF, Grenoble	D22, ESRF, Grenoble
Beam geometry	2 x 0.6 mm^2	2 x 0.6 mm^2	2 x 0.6 mm^2	Point	Pin-hole	Pin-hole
Wavelength (Å)	1.5	1.5	1.5	1.542	5.87	5.87
q range ($Å^{-1}$)	0.007 – 0.63	0.007 – 0.63	0.007 – 0.63	0.009 – 0.68	0.02 – 0.35	0.02 – 0.35
Exposure time (s)	120 (8 x 15)	120 (8 x 15)	120 (8 x 15)	7200 (8 x 900)	3600	10800
Concentration range (mg ml^{-1})	1.0 – 10.0	1.0 – 5.0	1.0 – 5.0	8.0 – 16.0	5.0	5.0
Temperature (K)	288	288	288	288	288	288
Structural parameters						
R_g (Å) from $P(r)$	23.4 ± 0.1	26.7 ± 0.1	37.7 ± 0.1	23.1 ± 0.1	36.4 ± 2.0	37.4 ± 2.0
R_g (Å) from Guinier	22.9 ± 0.1	25.0 ± 0.1	34.3 ± 0.9	23.1 ± 0.2	33.7 ± 0.7	34.3 ± 2.1
D_{max} (Å)	77.5	99	130	74.0	120 ± 10	120 ± 10
Porod volume estimated ($Å^3$)	29920	26930	72450	46180	N.D.	N.D.
Molecular-mass determination						
Molecular mass from Porod Volume (kDa)	22.7	22.6	-	-	-	-
Theoretical mass from sequence (kDa)	22.4	22.7	45.1	26.3	45.1	45.1
Software employed						
Primary data reduction	SASFLOW	SASFLOW	SASFLOW	SAXSLab	Rnils, Spolly	Rnils, Spolly
Data processing	PRIMUS	PRIMUS	PRIMUS	PRIMUS	PRIMUS	PRIMUS

Statistics according to Jacques et al [1].

Supplementary Table 3

DNA primers used in this work

Primer name	Primer sequence (5’ to 3’)
A1_1_for	TGATCCATGGGCTCTAAGTCAGAGTCTCCTAAAGAGC
A1_94_for	TGATCCATGGATTCTCAAAGACCAGGTGCCCAC
A1_97_rev	TCATGGTACCTTATCTTTGAGAATCTTCTCTGGAG
A1_196_rev	TCATGGTACCTTATCGACCTCTTTGGCTGGATGAAGC
E66C_for	GCCACTGTGTGCGAGGTGGATGCAGCTATGAATGCAAGGCCACAC
E66C_rev	CACCTCGCACACAGTGGCATATGTGACAAACCCAAAGCCCCTGG
R75E_for	ATGAATGCAGAGCCACACAAGGTGGATGGAAGAGTTGTGG
R75E_rev	GTGTGGCTCTGCATTCATAGCTGCATCCACCTCCTCCAC
R88E_for	GGAAGAGTTGTGGAACCAAAGGAGGCTGTCTCCAGAGAAGATTCT
R88E_rev	AGAATCTTCTCTGGAGACAGCCTCCTTTGGTTCCACAACTCTTCC
R88A_for	AAGAGTTGTGGAACCAAAGGCAGCTGTCTCCAGAGAAGAT
R88A_rev	ATCTTCTCTGGAGACAGCTGCCTTTGGTTCCACAACTCTT
SHAPE1	GCACTCAACATCAGCAGGCCCTGCAC
SHAPE2	CTATATACTTGCTTGGCTTG
SHAPE3	GACCTGCAGGCGGCCGCG

Supplementary Figure 1

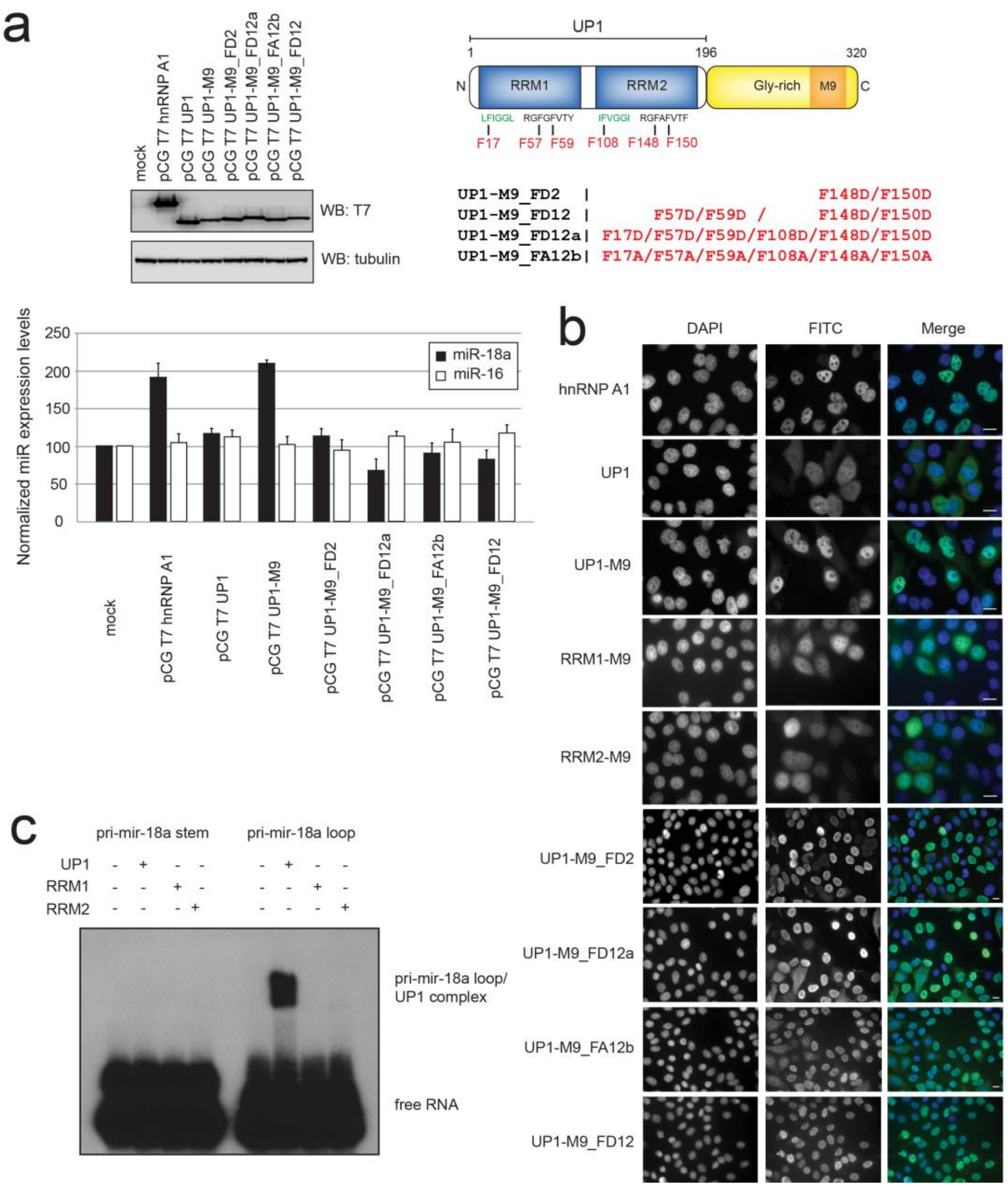

Supplementary Figure 1 – RNA binding and subcellular localization of hnRNP A1

(**a**) Effect of transiently transfected epitope-tagged hnRNP A1, UP1, UP1-M9 and UP1-M9 proteins harboring mutations in conserved Phe residues within RNP motifs in RRM1 and/or RRM2 domains in the processing of pri-mir-18a in HeLa cells in culture. The mutations are depicted on the right and mapped onto the domain structure of hnRNP A1. Processing of pri-mir-16 was included as a control (white bars). The upper panel shows the expression level of each construct. The M9 sequence was included to direct the nuclear localization of the UP1, RRM1 and RRM2 constructs. Mutational analysis show that RNA-binding is required for the stimulating activity of hnRNP A1.
(**b**) Subcellular localization of epitope-tagged proteins transiently expressed in HeLa cells. The cells were transfected with plasmids encoding the indicated T7-tagged proteins and fixed at 24 hr post-transfection. The localization of the expressed proteins was determined by indirect immunofluorescence with anti-T7 monoclonal antibody and FITC-conjugated secondary antibody. Bar: 5 µm. (**c**) EMSA using UP1, RRM1 and RRM2 with pri-mir-18a loop and stem RNAs.

Supplementary Figure 2

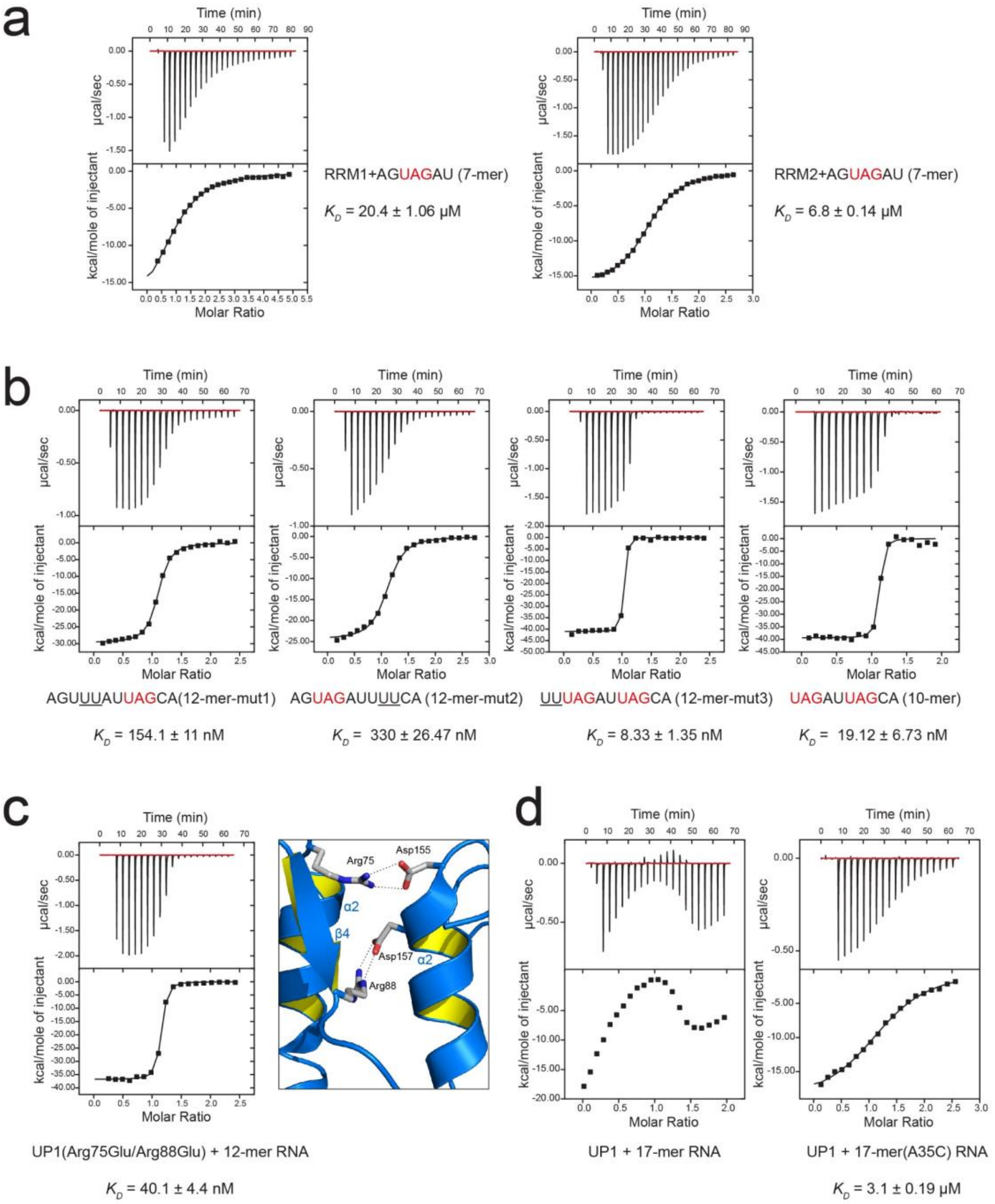

Supplementary Figure 2 – Isothermal titration calorimetry (ITC)

(**a**) ITC profiles of interaction of RRM1 and RRM2 with the 7-mer RNA. The UAG motifs are shown in red. (**b**) ITC profile of UP1 interaction with RNAs containing mutations in the UAG motifs. The UAG motifs are shown in red and the mutated nucleotides are underlined. (**c**) Effect of UP1 salt-bridge mutations in binding to the 12-mer RNA. The two Asp-Arg salt-bridges at the domain-domain interface in UP1 are shown. (**d**) ITC profile of UP1 interaction with the 17-mer and 17-mer(A35C) stem-loop RNAs. Binding of UP1 to the 17-mer RNA cannot be explained by a simple one-site binding model.

Supplementary Figure 3

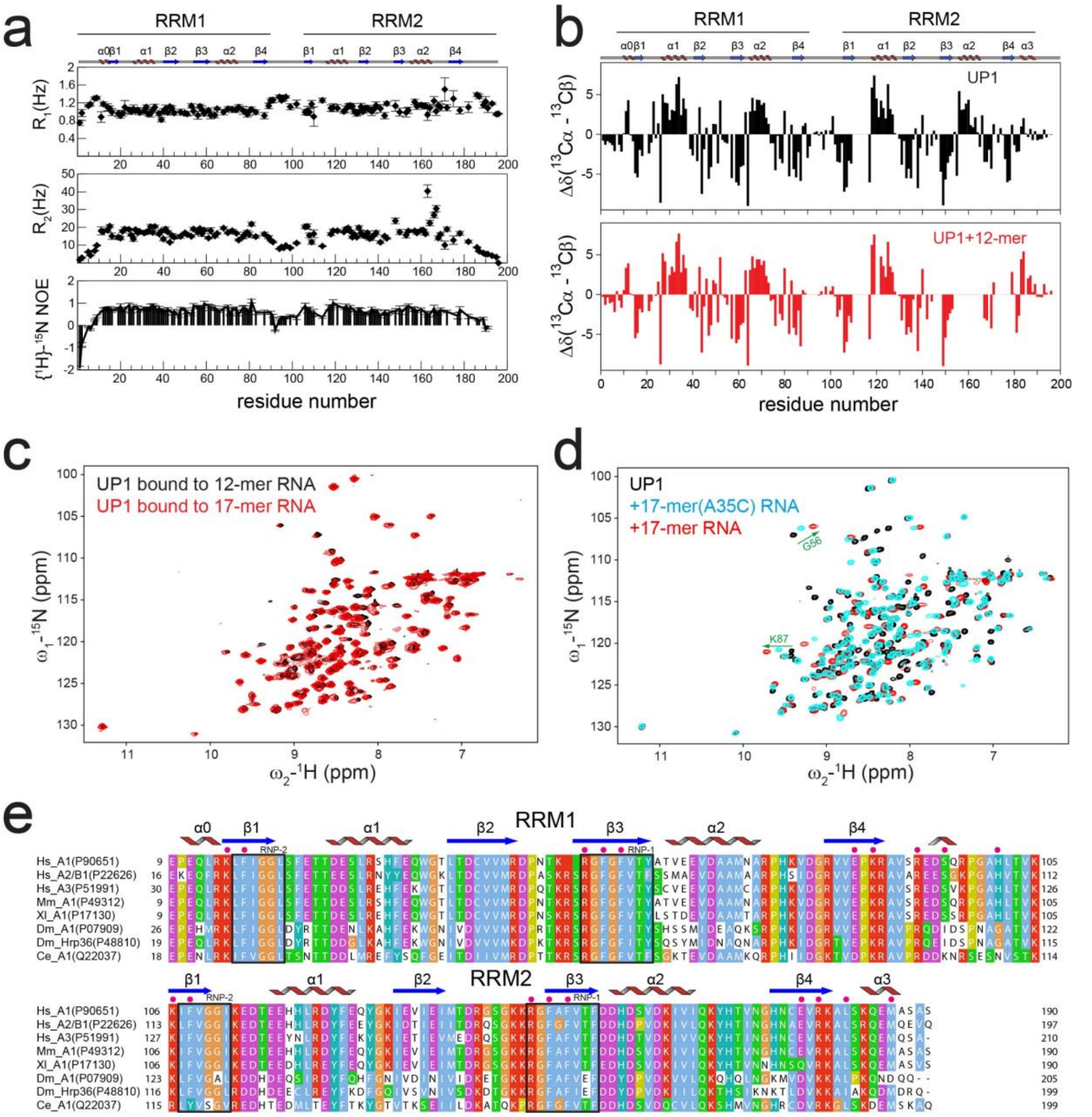

Supplementary Figure 3 – NMR data

(**a**) NMR relaxation data of UP1.^{15}N R_1, R_2 and heteronuclear {^{1}H} -^{15}N NOE values vs. residue number. Secondary structure elements are shown on top. Gaps in the data are due to overlapped or unassigned peaks. (**b**) Secondary chemical shifts of UP1 free (black, top) and bound to the 12-mer RNA (red, bottom). Secondary structure elements are shown above the plot. (**c**) Overlay of ^{1}H, ^{15}N HSQC spectra of UP1 free (black) and bound to the 7-mer RNA (red). Selected residues undergoing chemical shift changes and line-broadening upon RNA binding are labeled in green and black, respectively. (**d**) Overlay of ^{1}H, ^{15}N HSQC spectra of UP1 free (black) and bound to the 17-mer(A35C) (cyan) and 17-mer (red) RNAs. Two selected residues showing chemical shift perturbations are labeled in green. (**e**) Multiple sequence alignment of hnRNP A1. The sequence of the C-terminal Gly-rich tail is not shown. Secondary structure elements are indicated on top. Conserved RNP-1 and RNP-2 motifs are indicated by black boxes. Selected residues involved in interaction with the 12-mer RNA are marked by magenta circles. The UniProt accession code is indicated for each sequence. (Hs = *Homo sapiens*, Mm = *Mus musculus*, Xl = *Xenopus laevis*, Dm = *Drosophila melanogaster*, Ce = *Caenorhabditis elegans*).

Supplementary Figure 4

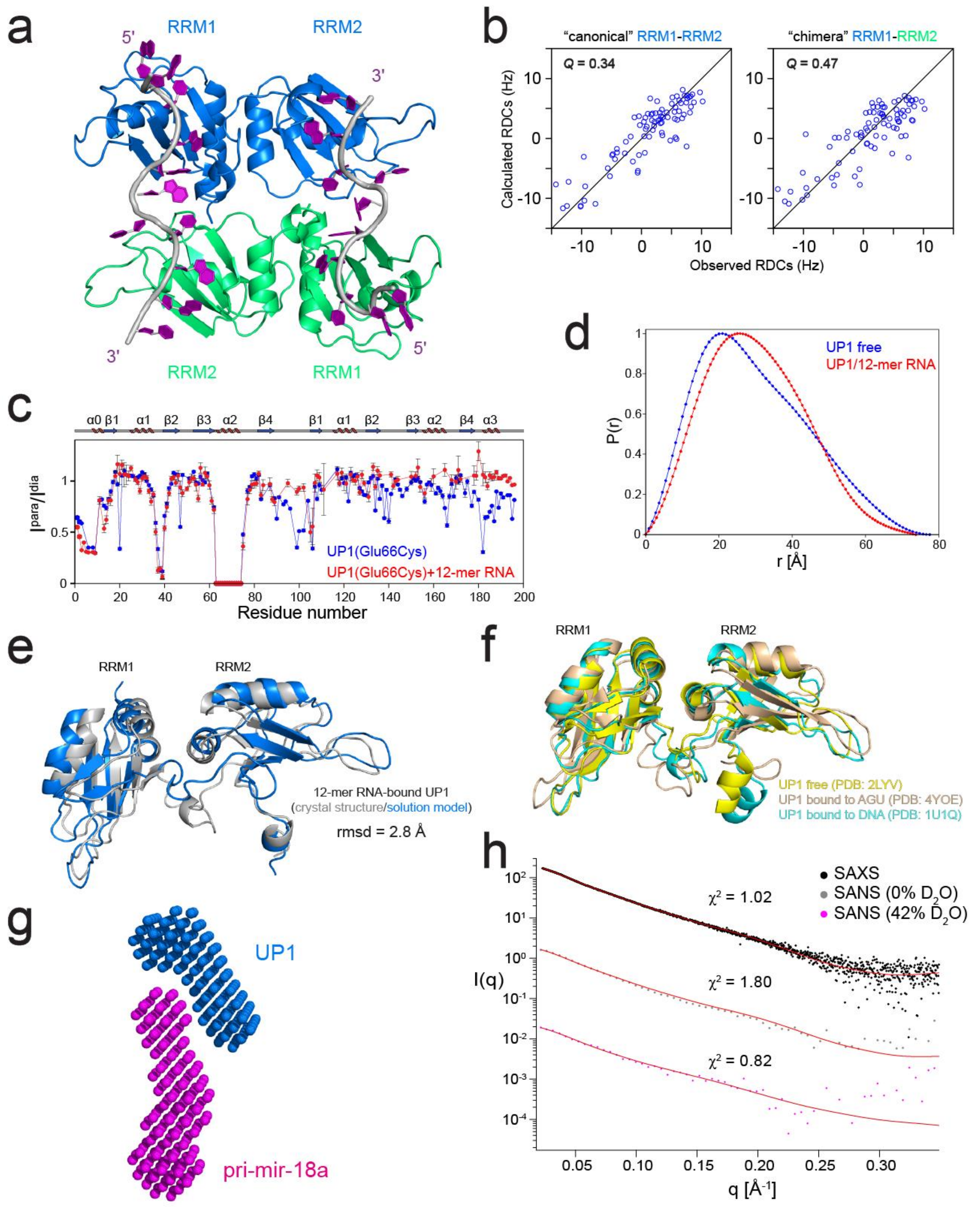

Supplementary Figure 4 – Crystal structure, NMR and small angle scattering data

(**a**) Crystal structure of the UP1/12-mer RNA complex at 2.5 Å resolution. The structure shows 2:2 (protein:RNA) stoichiometry and 5’→3’ directionality of the RNA, where the 5’-end of each RNA strand is recognized by RRM1, whereas the 3’-end binds to RRM2 from a second UP1 molecule. The two RNA chains are anti-parallel to each other. The two UP1 molecules in the asymmetric unit are colored differently. In this arrangement, the two UP1 molecules form a dimer mediated by

intermolecular contacts involving Glu11 in α0, Asp94 in the inter-RRM linker and four residues from RRM2 (Ile164, Lys166, Tyr167 and His173). The contacts at the dimer interface are the same as described for the UP1-DNA complex[2] and include hydrogen bonding (Glu11-His173 and Tyr167-Ile164) and a salt bridge between Asp94 and Lys166. (**b**) Correlation of experimental NMR RDC data for the RRM domain arrangements in the UP1/12-mer RNA complex in solution with those predicted from the two possible RRM1/RRM2 domain arrangements seen in the 2:2 crystal structure. (**c**) Paramagnetic relaxation enhancement (PRE) data of UP1 free (blue) and bound to 12-mer RNA (red) plotted as peak intensity ratios in the paramagnetic (I^{para}) and diamagnetic (I^{dia}) states. The spin-label is attached to a cysteine at position 66. Secondary structure elements of UP1 (in the RNA-bound form) are shown above the plot. PREs in the linker and C-terminal region of the free protein are due to flexibility. (**d**) SAXS-derived pairwise distance distribution for UP1 free and bound to the 12-mer RNA (see Table S2). (**e**) Comparison of domain arrangements in the 2:2 crystal structure of the UP1:12-mer RNA and the 1:1 model derived from the NMR and SAXS data. The overall arrangement of the tandem RRM domains is very similar as indicated by a backbone coordinate RMSD of 2.8 Å. (**f**) Domain arrangements in previously reported structures of UP1 free (NMR structure PDB: 2LYV)[3], UP1 bound to three nucleotides (crystal structure PDB: 4YOE)[4] and UP1 bound to DNA (crystal structure PDB:1U1Q)[5] are all very similar. (**g**) Ab initio bead model of the UP1/pri-mir-18a complex obtained using MONSA[6]. (**h**) Experimental and calculated SAXS/SANS intensity profiles of the UP1/pri-mir-18a complex are shown as dots and red lines, respectively.

Supplementary Figure 5

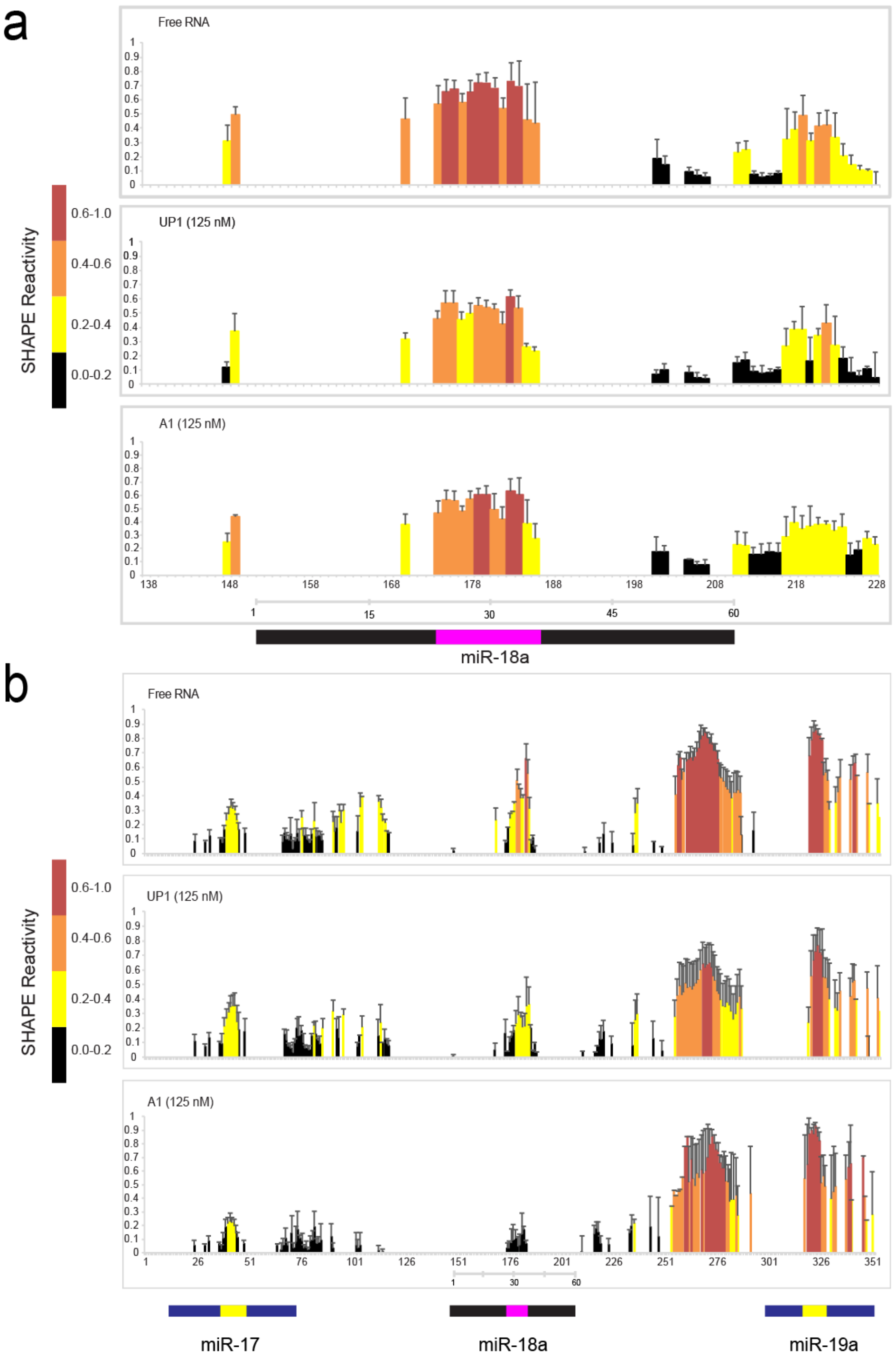

Supplementary Figure 5 – SHAPE of pri-mir-18a and the pri-mir-17-18a-19a cluster

(**a**) RNA SHAPE reactivity of pri-mir-18a as a function of nucleotide position for the free RNA and upon addition of purified UP1 or hnRNP A1 are depicted (top to bottom). (**b**) RNA SHAPE reactivity of pri-mir-17-18a-19a as a function of nucleotide position for the free RNA and upon addition of purified UP1 or hnRNP A1 are depicted (top to bottom). Bars are colored according to reactivity. Residue numbers refer to pri-mir-17-18a-19a, i.e. U152 in pri-mir-17-18a-19a corresponds to U1 in pri-mir-18a (71-mer), whereas the 12-mer sequence from the pri-mir-18a (71-mer) terminal loop (A27-A38) corresponds to A178-A189 in pri-mir-17-18a-19a. Boxes below the panels indicate the mature miRNA (blue) and pri-miRNA terminal loop (yellow/magenta) of miR-17, miR-18a and miR-19a.

Supplementary Figure 6

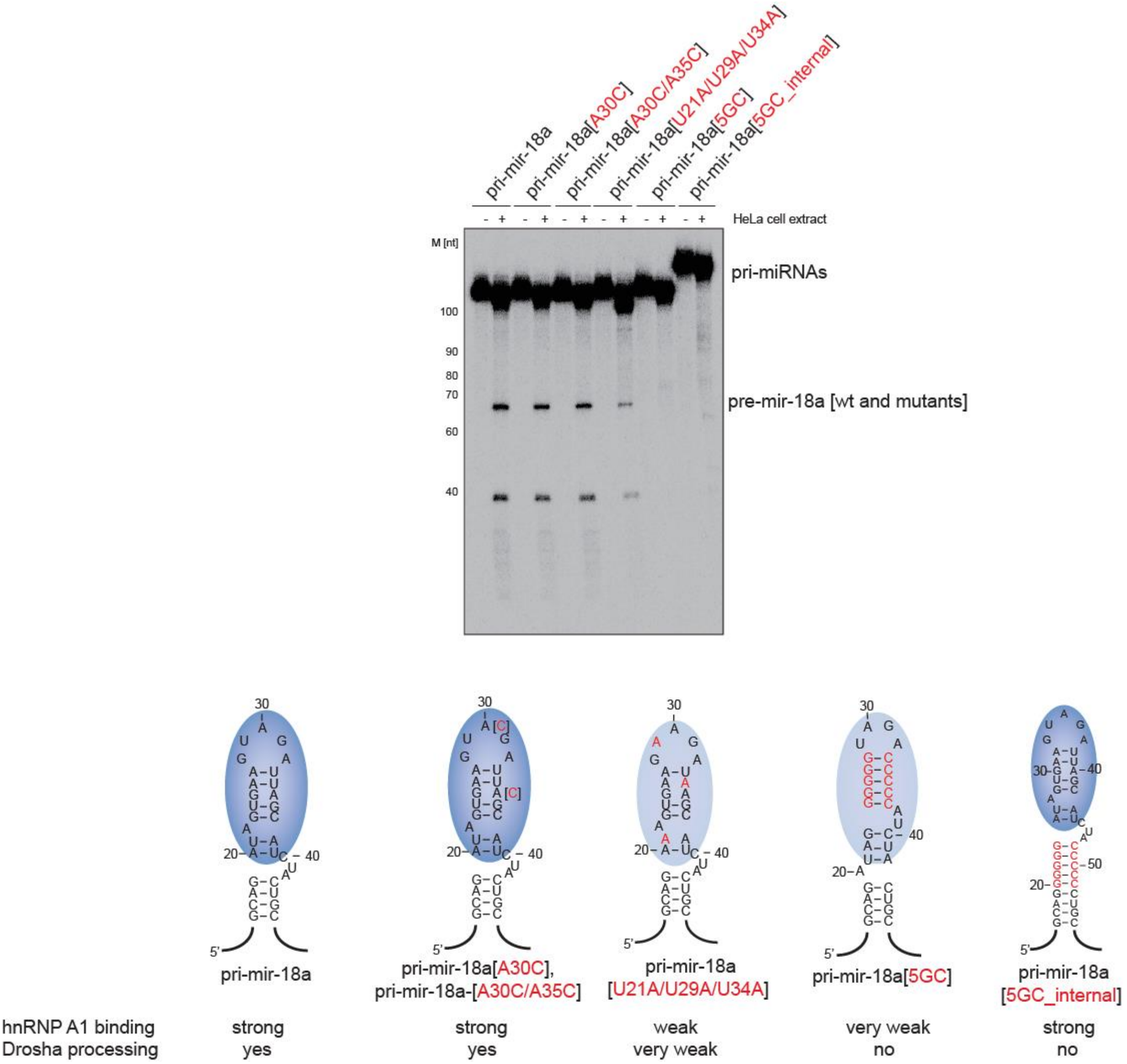

Supplementary Figure 6 – Terminal loop mutants in pri-mir-18a affect its processing

Isolated pri-miR-18a transcripts with point mutations introduced in Fig. 6 were subjected to *in vitro* processing assays. Wild type pri-mir-18a, pri-mir-18a[A30C] and pri-mir-18a[A30C/A35C] are processed with similar efficiency. Processing of pri-mir-18a[U21A/U29A/U34A] is noticeably reduced, whereas processing of pri-mir-18a[5GC] and pri-mir-18a[5GC_internal] is undetectable.

Supplementary Figure 7 – uncropped gel images

Figure 1

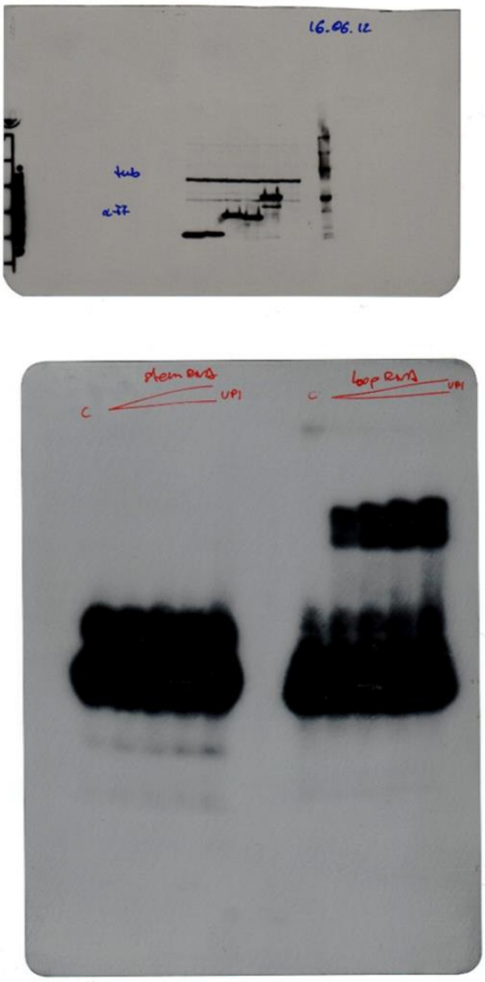

Figure 5

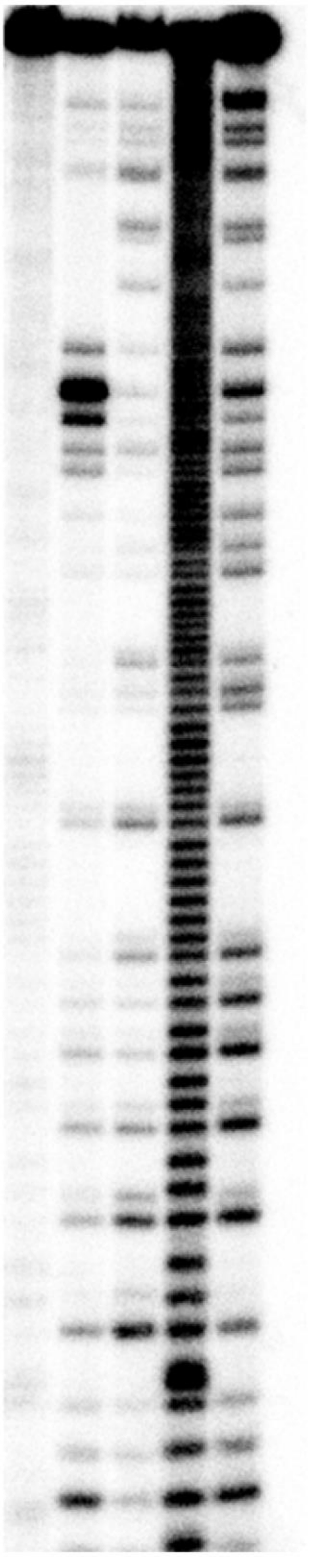

Figure 6

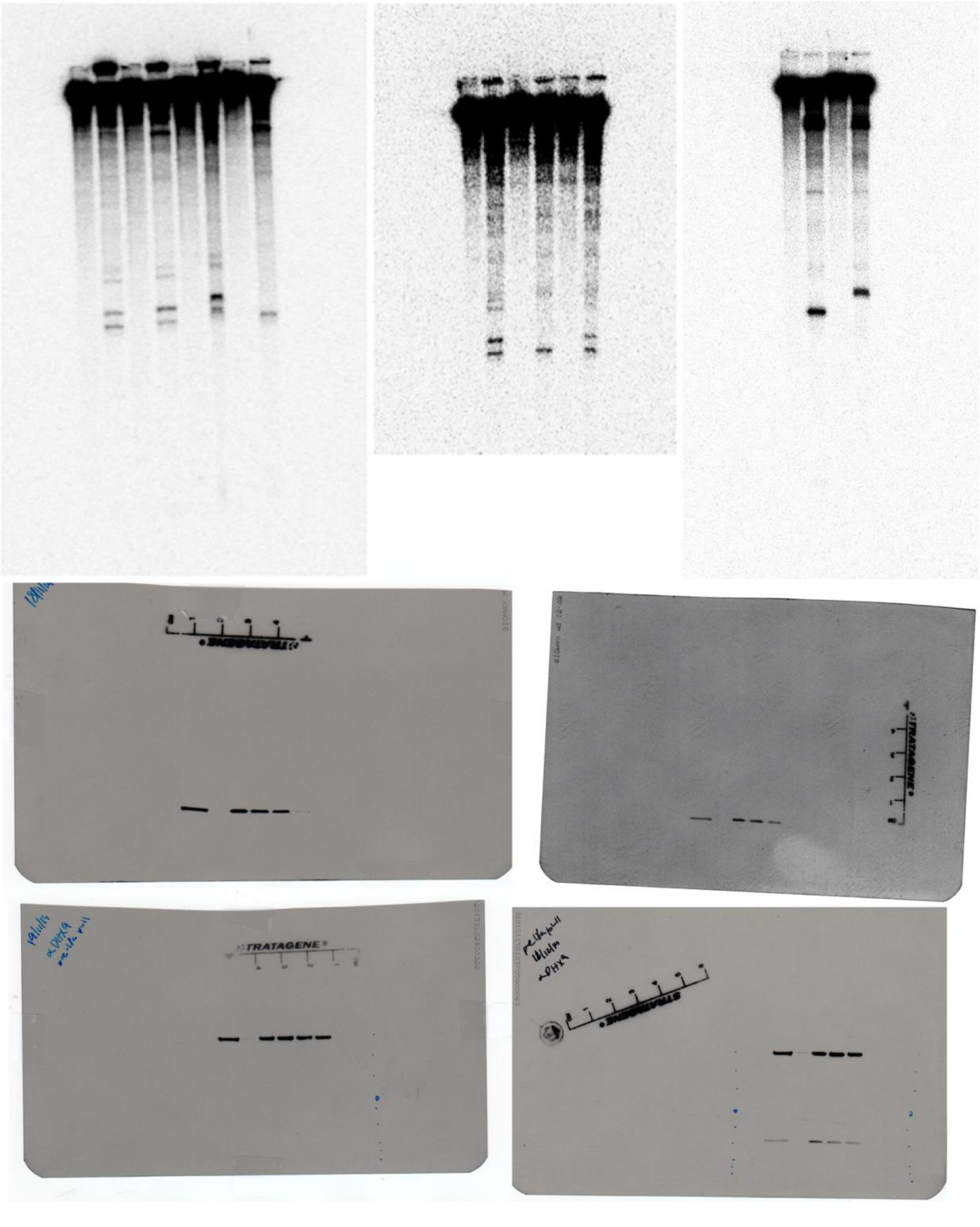

Figure 7

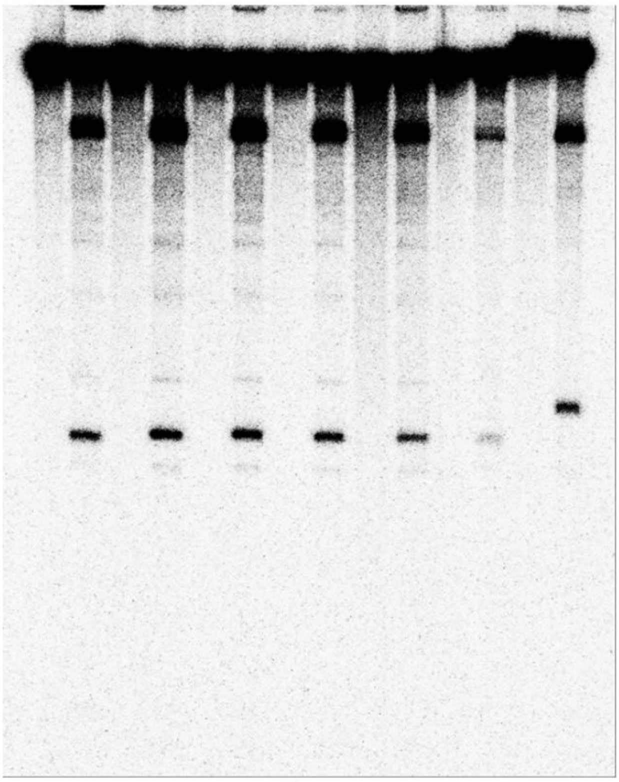

Supplementary Figure 6

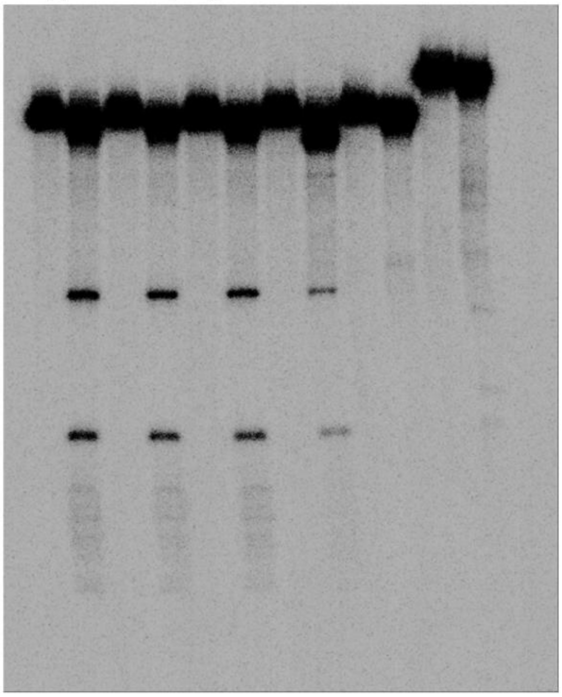

Supplementary Figure 1

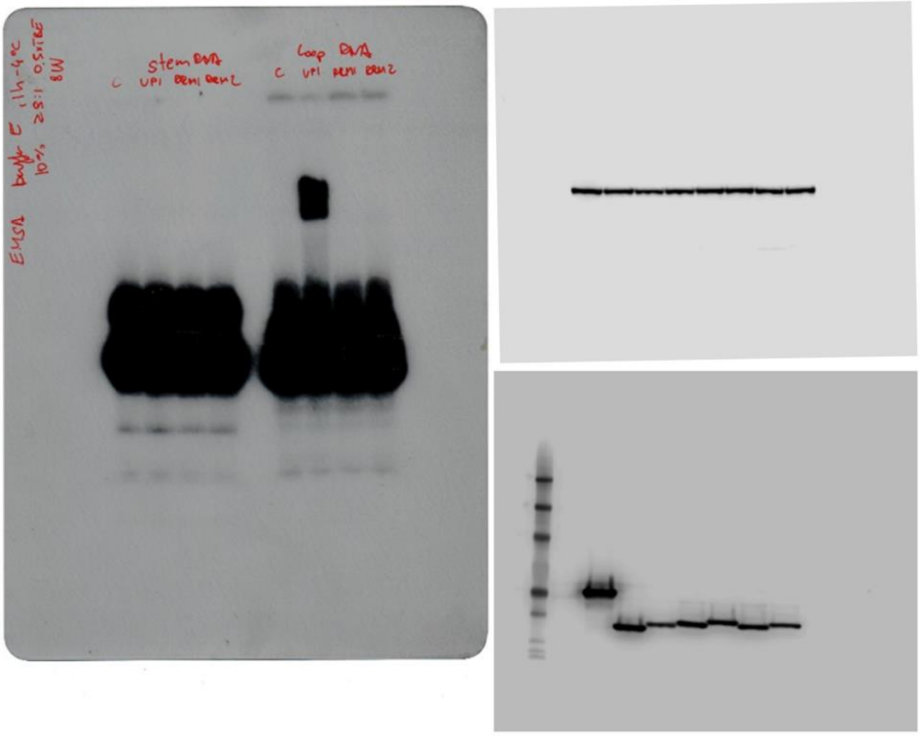

Supplementary References

1. Jacques, D.A., Guss, J.M., Svergun, D.I., Trewhella, J. Publication guidelines for structural modelling of small-angle scattering data from biomolecules in solution. *Acta Cryst. D Biol. Cryst.* **68**, 620-626 (2012).

2. Ding, J., *et al.* Crystal structure of the two-RRM domain of hnRNP A1 (UP1) complexed with single-stranded telomeric DNA. *Genes Dev.* **13**, 1102-1115 (1999).

3. Barraud, P., Allain, F.H. Solution structure of the two RNA recognition motifs of hnRNP A1 using segmental isotope labeling: how the relative orientation between RRMs influences the nucleic acid binding topology. *J. Biomol. NMR* **55**, 119-138 (2013).

4. Morgan, C.E., *et al.* The First Crystal Structure of the UP1 Domain of hnRNP A1 Bound to RNA Reveals a New Look for an Old RNA Binding Protein. *J. Mol. Biol.* **427**, 3241-3257 (2015).

5. Myers, J.C., Shamoo, Y. Human UP1 as a model for understanding purine recognition in the family of proteins containing the RNA recognition motif (RRM). *J. Mol. Biol.* **342**, 743-756 (2004).

6. Svergun, D.I. Restoring low resolution structure of biological macromolecules from solution scattering using simulated annealing. *Biophys. J.* **76**, 2879-2886 (1999).
